# Association of GWAS‐supported noncoding area loci rs404860, rs3117098, and rs7775228 with asthma in Chinese Zhuang population

**DOI:** 10.1002/jcla.23066

**Published:** 2019-10-12

**Authors:** Si‐Qiao Liang, Jing‐Min Deng, Xuan Wei, Zhang‐Rong Chen, Mei‐Ling Yang, Hua‐jiao Qin, Jian‐quan Zhang, Zhi‐yi He

**Affiliations:** ^1^ Department of Respiratory and Critical Care Medicine First Affiliated Hospital of Guangxi Medical University Nanning China

**Keywords:** asthma, GWAS‐supported, noncoding area loci, Zhuang population

## Abstract

**Background:**

Asthma is a complicated and polygenic inheritance disease, and its prevalence increases worldwide. Recent genome‐wide association studies (GWASs) identified a significant association of single nucleotide polymorphism with asthma in the Japanese population. This study aimed to examine the association of GWAS‐supported noncoding area loci, namely rs404860, rs3117098, and rs7775228, with asthma in Chinese Zhuang population.

**Methods:**

A case‐control study involving 223 individuals, comprising 123 patients with asthma and 100 healthy controls, was conducted. Genotypes were determined by polymerase chain reaction (PCR)/ligase detection reaction assay. The association between gene polymorphisms and asthma risk was calculated by logistic regression analysis using different genetic models through comparisons of alleles (A vs a), homozygote genotypes (AA vs aa), heterozygote genotypes (Aa vs aa), dominant models (AA+Aa vs aa), and recessive models (AA vs. Aa+aa).

**Results:**

The distribution of the genotype frequency of rs3117098 was statistically different between the case and control groups. For rs3117098, significant associations were observed through comparisons of alleles (OR: 1.832, 95% CI: 1.048‐3.204, *P* = .034) and dominant models (OR: 2.065, 95% CI: 1.001‐4.260, *P* = .050). The statistical analysis showed no significant difference for loci rs404860 and rs7775228 between patients with asthma and controls.

**Conclusion:**

rs3117098 may be the risk factor for asthma in Chinese Zhuang population.

## 1 INTRODUCTION

Asthma is a disease characterized by chronic airway inflammation and recurring respiratory symptoms, such as cough, chest tightness, shortness of breath, wheezing, and variable expiratory airflow limitation. Symptoms and airflow limitation characteristically vary over time in terms of intensity. Asthma also affects 1%‐18% of population in different countries, and its prevalence increases in many countries.^1^ Hospitalizations and deaths from asthma have declined in various areas, but the cost of asthma treatment remains a burden.

The etiology of asthma remains unclear. Asthma is a complicated and polygenic inheritance disease; familial aggregation and genetic predisposition are significant peculiarities of asthma.^2^, ^3^, ^4^, ^5^ Thus, the use of molecular genetic methods to explore the etiology and pathogenesis of asthma has become a research hotspot worldwide. Given the development of human genome haplotype plan data, the reduction in the costs of genome‐wide single nucleotide polymorphism (SNP) typing chip, and the emergence of new statistical methods and software, genome‐wide association study (GWAS) opens a new chapter in determining the genetic factors of complex diseases. GWAS, including advanced computational methods and rigorous replication, is a new platform for identifying genetic variations in complex polygenic disorders.^6^

Hirota et al^7^ conducted GWAS and replication study consisting of 7171 subjects with adult asthma and 27 912 controls in Japanese population; their results identified several candidate loci that are associated with susceptibility to adult asthma. Three GWAS‐positive mutations are located in noncoding areas, namely rs404860 (risk allele A), rs3117098 (risk allele G), and rs7775228 (risk allele A). SNP rs404860 is located in the *NOTCH4* gene in the major histocompatibility complex region, rs3117098 is located in the *BTNL2* gene (a member of the immunoglobulin superfamily), and rs7775228 is located in the *HLA‐DQA2* gene; these genes are inflammation‐related genes that may be involved in asthma development. Noncoding regions also regulate the expression of structural genes, which may be processed into various interfering RNAs to regulate the expression of other genes during transcription; these changes result in the corresponding genes being turned on, off, active, or inactive, thereby affecting the body's shape, development, and disease susceptibility.^8^ Therefore, noncoding region SNPs that are located in inflammation‐related genes should be studied in relation to incidence of asthma in Guangxi, China.

Several GWAS‐supported SNPs have been replicated in different independent studies, but the results are inconsistent. The asthma susceptible gene in Chinese minority has also been poorly studied. Guangxi is part of the Zhuang autonomous region and has the largest minority population in China. The Zhuang population in Guangxi also maintains ancient traditions in terms of language, ethnic culture, and lifestyle. The living environment and climate differ from the habitats of other ethnic groups. Considering the genetic backgrounds and the varying interaction between genes and environment in different races, conducting genetic research in the Guangxi Zhuang population is important. A case‐control study that includes patients with asthma and healthy controls in Guangxi Zhuang population was conducted to evaluate the association between GWAS‐supported noncoding area loci, namely rs404860, rs3117098, and rs7775228, and asthma.

## 2 MATERIALS AND METHODS

### 2.1 Study participants

A total of 123 patients with asthma and 100 healthy controls were nonconsecutively recruited from Guangxi Zhuang autonomous region. All the participants were natives of Guangxi Province and unrelated to one another. These participants belong to the Zhuang ethnic minority and permanently reside in Guangxi (three generations or more). These participants were diagnosed with asthma by at least two respiratory physicians in accordance with the guidelines of the Global Initiative for Asthma.^9^ A self‐report questionnaire including general conditions, medical history, and family history was administered to the controls to identify healthy individuals. The controls were excluded if they had a history of asthma, rhinitis, or other allergic or lung diseases. Case and control groups were enrolled from the Department of First Affiliated Hospital of Guangxi Medical University, Guangxi, China, between February 2010 and August 2016. The study was approved by the Institutional Ethics Committee of Guangxi Medical University under the full consideration of the Helsinki Declaration of Human Rights. Written informed consent was obtained from all subjects.

### 2.2 DNA isolation and SNP genotyping

Genome was extracted from 2 mL of peripheral venous blood by using a DNA extraction kit (Tiangen, Shanghai, China) in strict accordance with laboratory procedures. The presence of the three noncoding SNPs (rs404860, rs3117098, and rs7775228) for asthma was analyzed. Primer design (Primer 3 Online), synthesis, and genotype test were performed by Shanghai BioWing Applied Biotechnology Company (http://www.biowing.com.cn/). The primer sequences of the three SNPs are presented in Table 1. All the three SNPs were genotyped using the PCR/ligase detection reaction assay.

**Table 1:** Primers of target genes used in the PCR

SNP	Forward	Reverse	PCR length
rs7775228	TTCTTGCAGGAGGAAAGGAA	TGCAAAGCCCCTTTATCATT	96
rs3117098	TTGCACAACATCTAAAGGTTAACTA	GGCCTTCACAAGCACAGACT	104
rs404860	GCACCTCTGTGGGCTATGAT	CCAGATACGGAGGCAAATCT	110

The target DNA sequences were amplified using a multiplex PCR method. The reaction course included initial denaturation for 2 minutes at 95°C, followed by 40 cycles of denaturation at 94°C for 30 seconds, annealing at 53°C for 90 seconds, extension at 65°C for 30 seconds, and a final extension at 65°C for 10 minutes. After the reaction, 2 µL of each product was run in a 3.0% agarose gel to determine whether the reaction was successful.

The ligation reaction for each subject was performed in a final volume of 10 μL, containing 1 μL of 1× NEB Taq DNA ligase buffer, 1 μL of 2 pmol/μL of each probe mix, 0.05 μL of Taq DNA ligase, 4 μL of ddH_2_O, and 4 μL of multi‐PCR product. The ligase detection reaction was performed at 95°C for 2 minutes followed by 40 cycles at 94°C for 15 seconds and 50°C for 20 seconds. The fluorescent LDR products were differentiated by sequencer PRISM 3730 (ABI). Approximately 5% of the DNA samples were selected randomly and retested under blind conditions to assess the quality of genotypic data. The concordance rate was 100%.

### 2.3 Statistical analysis

Measurement and enumeration data were analyzed using SPSS 16.0. Chi‐square (*χ* ^2^) test was used to determine whether the genotype frequency distribution of the control was in the Hardy‐Weinberg equilibrium (HWE) and evaluate differences in genotypic distribution between the two groups. The association between the three polymorphisms and risk of asthma was calculated by logistic regression analysis using different genetic models. The odds ratio (ORs) and 95% confidence interval (CI) values were calculated. The genetic models were defined as follows: a variant allele (A vs a), homozygote genotype (AA vs aa), heterozygote genotype (Aa vs aa), dominant model (AA+Aa vs aa), and recessive model comparisons (AA vs Aa+aa). *P ≤ *.05 (two‐tailed) was considered to indicate statistically significant difference.

## 3 RESULTS

### 3.1 Characteristics of study subjects

A total of 100 control subjects (52 men and 48 women; median age and range: 27.9 years, 22‐67 years) and 123 individuals suffering from asthma (50 men and 73 women; median age and range: 38.8 years, 18‐71 years) were recruited and genotyped for rs404860, rs3117098, and rs7775228. The basic characteristics of the study subjects are listed in Table 2.

**Table 2:** Basic characteristics of the individual

Characteristics		Group	Case (n = 123)	Control (n = 100)	*χ* ^2^/*t* value	*P* value
Gender		Male	50 (40.7%)	52 (52.0%)	2.863	.091
		Female	73 (59.3%)	48 (48.0%)		
Age			39.06 ± 12.33	28.34 ± 3.31	9.203	.000
Height (cm)			157.83 ± 7.58	163.15 ± 6.50	−5.229	.000
Weight (kg)			54.98 ± 10.31	55.48 ± 10.00	−0.337	.736
Exposure to tobacco smoke	Ever‐smoker		42 (34.1%)	24 (24.0%)	2725	.099
	Never‐smoker		81 (65.9%)	76 (76.0%)		
Occupation	Farmer		99 (80.5%)	82 (82.0%)	0.063	.801
	Non‐farmer		24 (19.5%)	18 (18.0%)		
Exposure to allergens	Yes		20 (16.3%)	14 (14.0%)	0.218	.641
	No		103 (83.7%)	86 (86.0%)		
Co‐morbidities (atopic dermatitis)	Yes		30 (24.4%)	0 (0.0%)	28.181	<.001
	No		93 (75.6%)	100 (100.0%)		
Severity of asthma	Mild		46 (37.4%)	0 (0.0%)	NA	NA
	Moderate		32 (26.0%)	0 (0.0%)		
	Severe		45 (36.6%)	0 (0.0%)		

Abbreviation: NA, not available.

### 3.2 HWE test

Three SNPs, namely rs404860, rs3117098, and rs7775228, were detected in the Guangxi Zhuang population. The results showed that the genotypes were found in all SNPs. Genotyping analyses revealed that rs404860 and rs3117098 were distributed in HWE (*P* > .05) in the control group. However, the distribution of rs7775228 was not in HWE (*P* < .05) in the control group.

### 3.3 Polymorphism of the three SNPs in Zhuang population

The distribution of the genotype frequency of rs3117098 was statistically different between the case and control groups, as shown by *χ* ^2^ test (*P* < .05, Table 3).

**Table 3:** The genotype and allele frequencies of the three SNPs

SNPs	Genotype	Case (n = 123)	Control (n = 100)	*χ^2^* value	*P* value
rs7775228	TT	82 (67.70)	64 (66.00)	1.618	.445
	CT	33 (27.30)	24 (24.70)		
	CC	6 (5.00)	9 (9.30)		
rs3117098	TT	46 (38.00)	55 (56.70)	10.333	.006
	CT	56 (46.30)	37 (38.10)		
	CC	19 (15.70)	5 (5.20)		
rs404860	TT	58 (47.50)	47 (47.00)	0.335	.846
	CT	50 (41.00)	39 (39.00)		
	CC	14 (11.50)	14 (14.00)		

### 3.4 Association of the three SNPs in Guangxi Zhuang population with asthma

Logistic regression was used to assess the association between SNPs and asthma in Guangxi Zhuang, and the effects of gender, age, height, weight, exposure to tobacco smoke, occupation, and exposure to allergens were adjusted. The results of the three SNPs in different genetic models are shown in Table 4. For rs3117098, significant associations were observed using allele (OR: 1.832, 95% CI: 1.048‐3.204, *P* = .034) and dominant model comparisons (OR: 2.065, 95%CI: 1.001‐4.260, *P* = .050). The statistical analysis showed no significant difference for rs404860 and rs7775228 in any of the comparisons between patients with asthma and controls.

**Table 4:** The contribution of the three SNPs to the risk of asthma in different comparisons

SNPs	Comparisons	OR	95% CI	*P* value	OR_adj_	95% CI_adj_	*P* value_adj_
rs7775228	C/T	0.827	0.516, 1.324	.428	0.955	0.507, 1.797	.886
	CC/TT	0.520	0.176, 1.538	.237	0.712	0.157, 3.229	.659
	CT/TT	1.073	0.578, 1.993	.823	1.210	0.527, 2.776	.653
	CC+CT/TT	0.922	0.523, 1.627	.780	1.083	0.509, 2.306	.836
	CC/CT+TT	0.510	0.175, 1.487	.217	0.661	0.149, 2.929	.585
rs3117098	C/T	1.986	1.308, 3.017	.001	1.832	1.048, 3.204	.034
	CC/TT	4.543	1.574, 13.116	.005	3.110	0.882, 10.970	.078
	CT/TT	1.810	1.023, 3.202	.042	1.882	0.873, 4.059	.107
	CC+CT/TT	2.135	1.239, 3.679	.006	2.065	1.001, 4.260	.050
	CC/CT+TT	3.427	1.230, 9.549	.018	2.372	0.708, 7.951	.162
rs404860	C/T	0.933	0.626, 1.389	.732	0.624	0.361, 1.077	.090
	CC/TT	0.810	0.352, 1.867	.621	0.581	0.182, 1.852	.359
	CT/TT	1.039	0.588, 1.834	.895	0.522	0.235, 1.160	.111
	CC+CT/TT	0.979	0.576, 1.662	.936	0.522	0.250, 1.090	.084
	CC/CT+TT	0.796	0.360, 1.760	.573	0.732	0.253, 2.118	.565

Abbreviation: adj: After adjusting for gender, age, height, and weight, exposure to tobacco smoke, occupation, exposure to allergens.

## 4 DISCUSSION

To our knowledge, this study is the first to report the association of the polymorphisms of GWAS‐supported noncoding area loci, namely rs404860, rs3117098, and rs7775228, with asthma in the Chinese Zhuang population. The results indicated that rs3117098 was significantly associated with asthma susceptibility in the Chinese Zhuang population.

SNP rs3117098 is located within the *BTNL2* intron. BTNL2 is a member of the immunoglobulin superfamily, and butyrophilin proteins share the same sequence homology with B7 molecules.^10^ The B7 family is the second signal of T‐cell activation, that is, this family is a costimulatory molecule that regulates T‐cell activation and tolerance. Thus, BTNL2 is the first member of the butyrophilin family that regulates T‐cell activation, which has implications in immune diseases and immunotherapy. According to the homology of B7‐1, *BTNL2* is a costimulatory molecule that is involved in T‐cell activation.^11^ The occurrence of asthma is related to T‐cell imbalance. Therefore, the occurrence of asthma may be related to *BTNL2*. Konno et al^12^ found that the *BTNL2* gene is a candidate gene responsible for the pathogenesis of Der f‐specific IgE responsiveness. At present, no research about *BTNL2* gene polymorphism and asthma has been reported locally and internationally.

SNP rs404860 is located in the intron area of the *NOTCH4* gene, which was first discovered in *Drosophila* in 1919. The NOTCH signaling pathway is an important signal transduction pathway that affects cell development, differentiation, proliferation, and apoptosis.^13^ The NOTCH signaling system is also important in the differentiation and activation of peripheral effector T cells. Asthma is associated with IgE‐mediated allergic inflammation, and T lymphocytes and eosinophils are the main effector cells. Therefore, NOTCH signal may be associated with the pathogenesis of asthma. The correlation of *NOTCH4* gene polymorphism and asthma has not yet been reported. In the present study, the distribution of rs7775228 was not in HWE in the control group. Small sample size and nonrandom sample may have generated a type I error in analysis. An alternative explanation is a random chance. A review of published association studies by Xu et al^14^ indicated that 12% of the tested SNPs are inconsistent with HWE in control subjects.

SNP rs7775228 is located between the upstream regions of *HLA‐DQA2* and *HLA‐DQB1*. *HLA‐DQA2* and *HLA‐DQB1* encode HLA class II molecules, including *HLA‐DQ* α and β chains, which are expressed in antigen‐presenting cells.^15^ HLA class II molecules play an important role in the immune system by presenting peptides derived from extracellular proteins.^16^ Nakajima et al^17^ conducted GWAS and replication study using 4800 Japanese subjects and found the association of rs7775228 with susceptibility to knee osteoarthritis. Ramasamy et al^18^ conducted a genome‐wide meta‐analysis and found that the HLA variant rs7775228 is strongly associated with grass sensitization but weakly associated with allergic rhinitis. To our knowledge, no study has investigated the relationship between rs7775228 polymorphism and asthma.

GWAS is a widely used tool for searching genetic factors in asthma but is not always straightforward in replicating the original GWAS results. Hirota et al^7^ reported the association of GWAS‐supported noncoding area loci rs404860, rs3117098, and rs7775228 with asthma. However, our study indicated that only rs3117098 polymorphism may increase the risk of asthma in the Chinese Zhuang population. One of the possible reasons for this result is the fact that different races have different genetic backgrounds and living environments. Another possible reason is the extremely sample size in this study.

Several limitations should be pointed out. First, our samples in the case group were mainly based on patients from the First Affiliated Hospital of Guangxi Medical University and not from other hospitals, thereby possibly generating an inevitable selection bias. Thus, further research with additional representative samples, including those from different hospitals, is needed. Second, this study was only conducted among participants from the Chinese Zhuang population. The genetic association results should be treated with caution when extrapolating to other populations because of ethnic heterogeneity. Third, although this study yielded several positive results, the potential biological function mechanisms have not been elucidated to date. This case‐control study only provides the hypothesis of the epidemiological etiology but fails to reveal how genetic polymorphism specifically affects the pathogenesis of asthma. In future studies, cell function and animal experiments should be conducted to explore the mechanism underlying the positive results.

In summary, our case‐control study failed to replicate the GWAS‐identified association of rs404860 and rs7775228 with asthma. However, significant findings showed that rs3117098 polymorphism was associated with asthma.

## CONFLICTS OF INTEREST

All authors report no conflicts of interest.
